# Predicting employee telecommuting preferences and job outcomes amid COVID-19 pandemic: a latent profile analysis

**DOI:** 10.1007/s12144-021-02496-8

**Published:** 2021-11-19

**Authors:** Cafer Bakaç, Jetmir Zyberaj, James C. Barela

**Affiliations:** 1 Chair of Psychology, TUM School of Management, Technical University Munich, Arcisstraße 21, D-80333 Munich, Germany; 2 Chair of Work and Organizational Psychology, Department of Psychology, University of Bamberg, Bamberg, Germany; 3 TUM School of Education, Technical University Munich, Munich, Germany

**Keywords:** Conscientiousness, Autonomy, Self-regulation, Extraversion, Telecommuting preferences, Latent profile analysis

## Abstract

In this two-study research using latent profile analysis (LPA), we investigated intra-individual combinations of conscientiousness, autonomy, self-regulation, and extraversion. Based on these combinations, we designed profiles and explored telecommuting preferences and job outcomes of employees during the COVID-19 pandemic. In Study 1, we recruited 199 participants (77 females, ages ranging from 18 to 65). Results of this study revealed three profiles. One profile scored high on all of the variables and displayed preferences for working on-site more than the other profiles. Additionally, this profile showed higher work engagement, job satisfaction, and perceived productivity than the other two profiles. To validate these findings, we conducted a second study with a sample of 492 participants (169 females; age ranged from 18 to 65). The results yielded five profiles, one scoring high on all of the variables. Similar to Study 1, this profile exhibited higher work engagement, job satisfaction, and perceived productivity than the other four profiles. Individuals in this profile preferred to work on-site compared to individuals in other profiles. Our findings add to the research demonstrating the importance of personality characteristics for telecommuting preferences and work-related outcomes.

**Supplementary Information:**

The online version contains supplementary material available at 10.1007/s12144-021-02496-8.

With the rapid advancements in telecommunication technology, more and more people use diverse alternatives of work. In a review paper, Spreitzer et al. (2017) reported that changes in the alternative work arrangements (i.e., the flexibility about where work takes place), has taken a sharp increase in the last 10 years, with many companies going completely virtual. For example, Katz and Krueger (2016) showed that between 2005 and 2015, workers in alternative work arrangements increased from 10.1% to 15.8%. The Global Workplace Analytics (2019, as cited in Molla, 2019) predicted that over 70% of the workforce will be working remotely (at least five days per month) in the next five years. Despite the implications yielded by various crises, according to Spreitzer et al. (2017), in addition to the innovations in technology, a firm’s flexibility and workers’ preferences are two of the main determinants of these new forms of work. They report that, in these new work arrangements, telecommuting (defined as the “remote work that uses computer technology to work from home or another location away from the office”, p. 478), is one important factor, especially influencing the preferences of workers in the new and globalized economy. Such preferences have been also confirmed by research revealing that over 50% of contract workers prefer independent work due to the freedom and flexibility it provides (Lincoln & Raftery, 2011). Telecommuting provides workers with more freedom and flexibility, offering many benefits, such as for work-family (Allen et al., 2013). These forms of work gain support by social theories, such as the social exchange theory (Blau, 1964), which indicates that features such as flexibility raise workers’ feelings of obligation towards their company, which in turn may enhance their work performance.

Telecommuting as a new form of work is reported to increase steadily and has been the subject of a growing body of research (Gajendran & Harrison, 2007; Lee, 2021; Raghuram et al., 2019; Spreitzer et al., 2017). Nevertheless, researching its state and implications during unusual contexts, such as COVID-19, is of special interest and significance. The United Nations (2020) and the World Health Organization (2020) report that COVID-19 has had a tremendous impact on people’s lives, including workplaces. There is already evidence that various organizational and individual factors could mitigate negative effects of COVID-19 on workplace experiences. Current research has shown that factors such as the quality of communication between members of an organization, as well as the individuals’ personal resources (i.e., psychological safety) play a role in one’s telecommuting experiences during the pandemic (e.g., Lee, 2021). Similarly, others have reported that factors such as one’s motivation (Mahmoud et al., 2020) or gender (Feng & Savani, 2020) are important factors that predict an employee’s telecommuting tendencies and preferences. In line with these findings, our current research extends the current literature by investigating the relationship of motivational and personality characteristics and employees’ telecommuting preferences in the context of COVID-19.

As opposed to the regular modes of work, telecommuting varies substantially from how employees perform their usual work, with different settings and usually from home (Allen et al., 2015). Findings suggest that telecommuting can yield both benefits and harms, for both employees and organizations (Barsness et al., 2005; Fonner & Roloff, 2010; Gajendran & Harrison, 2007; Raghuram et al., 2019).

In a meta-analysis, Gajendran and Harrison (2007) emphasized the lack of a theoretical framework for telecommuting consequences and benefits. Nevertheless, they reported that most research highlights three main conceptual themes in telecommuting literature, which are, broadly speaking, about the “psychological process or intervening mechanisms through which telecommuting has its effects” (p. 1525). These themes comprise (a) psychological control or perceived autonomy (i.e., employees’ perceived control over their work task; (b) telecommuting’s effects on the work-family interface (where empirical findings are reported as inconclusive); and (c) telecommuting’s potential for relational impoverishment at work (i.e., due to a weakening of the relationships among various stakeholders in organizations). The good news is that these themes have been empirically tested; the “bad” news is that no conclusive evidence has been provided so far. Gajendran and Harrison (2007) reported that despite the challenges and negative influences, “telecommuting is mainly a good thing” (p. 1535). They posited that telecommuting is associated with increased perceptions of autonomy and lower work-family conflict. The first position (i.e., perceptions of autonomy) is relatively well established, with research reporting that telecommuting offers a good sense of autonomy due to an expressed feeling of freedom and discretion, as well as due to the opportunity to spatially and psychologically distance interactions between supervisors and employees (Dubrin, 1991; Raghuram et al., 2001; Shamir & Salomon, 1985). On the one hand, the availability of information and communication technologies might make it difficult for employees to psychologically detach (Greenhaus & Beutell, 1985) as well as work longer hours than usual when telecommuting (Boswell & Olson-Buchanan, 2007). On the other hand, the flexibility that telecommuting yields, can also provide room for productivity and healthy work. For instance, employees can practice more segmentation at home and utilize the benefits of reducing commuting hours for family-related activities, which will enhance family boundaries and thus mitigate the negative effects of telecommuting (Greenhaus & Beutell, 1985). Finally, based on social presence theory (Short et al., 1976) and richness theory (Daft & Lengel, 1986), researchers have reported adverse effects of telecommuting in terms of the relationship among members of an organization (Harrison et al., 2000; Higa et al., 2000; Workman et al., 2003). The quality of relationships among members of an organization is affected mainly by alterations in the frequency and quality of interactions. When the frequency of interaction is reduced because of telecommuting, the quality of relationships among members becomes impaired, which in turn, might affect work-related outcomes. Similarly, McCloskey and Igbaria (2003) argued that employees who telecommute might perceive their supervisors as questioning their commitment. In addition, they also reported that managers might also fear the loss of control over their subordinates. These personal perceptions about the quality of the relationships between leaders and members in organizations is yet another example of the effects of telecommuting on the work-relationships and -outcomes.

Furthermore, telecommuting implications on work engagement, productivity, and job satisfaction have also been a target of research, with an increased interest over the past few years (Galanti et al., 2021; Ojo et al., 2021; Rudolph & Baltes, 2017; Sonnentag et al., 2008). For instance, Sonnentag et al. (2008) reported that one’s ability to psychologically detach or “switch off” from work during their out-of-work activities is a significant factor for employees’ engagement. As posited by early research (e.g., Greenhaus & Beutell, 1985), telecommuting challenges employees’ regular working hours (i.e., working longer hours), which in turn can hinder their ability to detach from work. Similarly, under the Job Demands-Resources (JD-R) model (Demerouti et al., 2001), previous research has noted the many effects of working conditions on employees’ productivity. Because telecommuting requires that employees adapt to new forms of working conditions (e.g., less social contact), it also implies direct effects on their productivity. For example, alluding to remote work as an important job demand, Galanti et al. (2021) reported that a lack of social interactions, difficulty to reconcile between the private and work environment, as well as difficulties of arranging work activities at home, are only some of the challenges that working from home yields for employees’ job productivity. Moreover, although a large number of studies have been conducted in relation to telecommuting implications on employees’ job satisfaction, its effects are rather ambiguous. One stream of research has reported the positive effects of working from home on job satisfaction by letting employees adjust their work tasks (Baltes et al., 1999) or meet non-work, family responsibilities (Riley & McCloskey, 1997). The other stream posits that these positive benefits may be outweighed by the lack of social interaction and increased isolation due to telecommuting (Cooper & Kurland, 2002; Golden & Veiga, 2005; Shapiro et al., 2002). For instance, the lack of interactions affects employees’ relationships with their coworkers or supervisors, which in turn might influence their job satisfaction.

Previous research has reported that employee personality variables can play a major role in mitigating the negative effects of telecommuting, as well as on employees’ job-related outcomes, such as productivity, work engagement, and job satisfaction (Blickle et al., 2015; Göllner et al., 2017; Smith et al., 2015). For example, among the Big Five personality traits, conscientiousness is reported as the best predictor of job performance (Barrick & Mount, 1991). Other research has posited that conscientiousness can indeed be improved, through such home-activities as homework (Göllner et al., 2017). Furthermore, self-regulation strategies have been shown to be very important for one’s persistence at work (e.g., Latham & Locke, 2007). Similarly, extraversion has been reported to be one of the most consistent personality predictors of leadership (e.g., Blickle et al., 2015), with these individuals preferring a flexible work environment (Clark et al., 2012). Moreover, the satisfying the need for autonomy has been reported to be important for job performance by increasing motivation, interest, engagement, and job satisfaction (Huyghebaert-Zouaghi et al., 2020; Morgeson & Humphrey, 2008). In line with these findings, we examined the intra-individual (i.e., within-person) combinations of conscientiousness, need for autonomy (henceforth as autonomy), self-regulation, and extraversion. In our research, we refer to these variables as CASE. Utilizing CASE, we conducted person-centered Latent Profile Analysis (LPA), aiming to create certain employee profiles and then investigate their telecommuting preferences (Spreitzer et al., 2017). In addition, we employed work engagement, job satisfaction, and perceived productivity as important work-related outcomes.

To report on conscientiousness and extraversion, we draw on Big Five theory and research (e.g., Clark et al., 2012; Haines et al., 2002; McCrae & Costa, 2003), while for self-regulation and need for autonomy we drew on various motivational theories and models, such as the Self-Determination Theory (SDT; Ryan & Deci, 2000) and Beal et al.’s (2005) model of self-regulation. A number of papers have reported on the benefits that Big Five features may play for employee job performance, as well as their telecommuting preferences (e.g., Clark et al., 2012; Haddon & Lewis, 1994). Based on previous research and theory reporting on the benefits of the Big Five for telecommuting, we selected only conscientiousness and extraversion. For example, the organizational fit theory (Judge & Cable, 1997; Ryan & Kristof-Brown, 2003) postulated that “individuals seek out situations that are congruent with their personalities, and empirical research supports this basic tenet of interactional psychology” (Judge & Cable, 1997, p. 364). A study by Clark et al. (2012) stated that extraverts could utilize benefits that telecommuting yields and “design offsite environments that allow them to spend more time with family and friends” (p. 33). Similarly, conscientiousness has been reported as another predictor of job performance, and characteristics of individuals high in conscientiousness (e.g., responsible, organized, self-disciplined; McCrae & Costa, 2003) have been linked with employees’ positive telecommuting attitudes because they might facilitate enacting work routines and be able to act independently on their work tasks (Haddon & Lewis, 1994; Haines et al., 2002). Thus, we expect that conscientiousness and extraversion might facilitate one’s transition from an office environment to telecommuting situations. Furthermore, drawing on the SDT (Ryan & Deci, 2000) we assume that the need for autonomy can be an important factor for employees’ behavioral regulation during telecommuting. According to SDT, autonomy (e.g., a strong desire to be one’s self agent; Deci & Vansteenkiste, 2004) plays a major role in one’s behavior regulation. Linking to their goals and values, autonomous individuals can experience and express a high level of regulation in challenging contexts, especially when there is an environment that “allows the person to feel competent, related, and autonomous” (Ryan & Deci, 2000, p. 74). Thus, we expect that employees with a high level of autonomy will show a greater preference for telecommuting, which will ultimately improve their work performance. Finally, based on Beal et al.’s (2005) model of self-regulation and previous empirical research, we predict that self-regulation will be another crucial personality factor to facilitate employee telecommuting, increasing their preferences towards working off-site. According to Beal et al.’s (2005) model, self-regulation at work is affected by three forces: regulatory resource, task attentional pull, and off-task attentional demands. The authors indicate that any strain from the environment is likely to have an impact on regulatory processes. Similarly, off-task attentional demands can also drain one’s resources by taking resources away from the main task. Thus, individuals need to have a high level of self-regulation skills in order to successfully manage demanding environments. However, this is where the task attentional pull can help individuals with their demanding environments. Beal and colleagues argue that task attentional pull can facilitate one’s functioning in demanding environments by helping individuals to focus on relevant and focal tasks, which will preserve their resources and mitigate the negative effects of strains coming from the environment. This way, we assume that self-regulation will play a positive role in employees’ telecommuting preferences.

With this research, we contribute to the literature and practice in several ways. Theoretically, drawing on motivational and personality research and theory, we provide further support to pertinent theories concerning the implications of our chosen constructs for telecommuting and job-related outcomes. Practically, our research provides manifold ramifications for both, individuals and organizations, at large. For example, by predicting preferences for working from home or working on-site based on the personality and motivational characteristics of employees, we provide information that companies can utilize to leverage and maximize the efficiency of their workforce. Using CASE variables, our profile analysis reveals the “type” of employee that would be most suited for telecommuting, especially in times of crisis and pandemics, such as COVID-19. In addition to telecommuting, our research contributes by investigating the relationships between the profiles revealed and important work-related outcomes (namely, work engagement, job satisfaction, and perceived productivity). Thus, by combining the “best” types of profile(s), predicting their telecommuting preferences, and correlating the type with work-related outcomes, we provide data to both individuals and organizations at large on the relevant implications. On the one hand, individuals (i.e., employees) can learn about the personality- and motivational-characteristics that could predict telecommuting preferences, as well as better job performance. On the other hand, organizations can use this information to prepare their workforces to excel in times of crisis (i.e., such as COVID-19).

## Latent Profile Analysis

Latent profile analysis is a probabilistic mixture modeling technique, used to identify a set of discrete latent classes of individuals by looking at their individual responses to a set of indicators (Tein et al., 2013). Thus, the main aim is to “identify types, or groups, of people that have different configural profiles of personal and/or environmental attributes” (Spurk et al., 2020, p. 2). Studies have reported that, when compared to traditional cluster analyses, probability-based mixture modeling is exceptionally effective for detecting latent taxonomies (e.g., Peel and McLachlan, 2000). Using a set of indicators, various numbers of profiles (constellations) are identified and, based on research aim, can be linked to various antecedents and outcomes. As a person-centered approach, LPA offers a robust and holistic approach for investigating the interaction of the different motivational and personality factors (Morin & Wang, 2016). Gabriel et al. (2015) reported that, as opposed to variable-approaches (predicting outcomes separately and across people), the “person-centered approaches allow researchers to understand how variables operate conjointly and within people to shape outcomes” (p. 865). Thus, differences in profiles that result from a person-centered approach can vary across various profile indicators (i.e., fit indices), both quantitatively (i.e., level) and qualitatively (i.e., shape), facilitating the identification of the distinct profiles of employees (Gabriel et al., 2015; Spurk et al., 2020). These variances provide powerful ways to gauge profiles’ absolute and relative standing on the respective profile indictors (Gabriel et al., 2015; Charzyńska, 2020; Chawla et al., 2020).

## The Present Research

Using the person-centered approach, in this two-study research we combined conscientiousness, autonomy, self-regulation, and extraversion (abbreviated at CASE), to identify “X” number of profiles. CASE variables were selected based on previous research (i.e., theoretically meaningful findings for profile correlates) and their implications for various work-related outcomes (Morin et al., 2011). With an exploratory approach, identifying the number of profiles was determined solely on the LPA criteria, using a set of variables (i.e., LPA indicators). Accounting for previous research findings, reporting on the significance of each of the CASE constructs for employees, we expected these variables to yield robust profiles. These profiles were then investigated for their telecommuting preferences and examined in terms of their correlations with work engagement, job satisfaction, and perceived productivity. We expected that at least one profile would score high on all CASE variables. Similarly, we predicted that individuals in this profile would be willing to telecommute more than other profiles. In addition, we expected that this profile would be positively correlated with work engagement, job satisfaction, and perceived productivity. Furthermore, we estimated an indirect effect of profiles on job satisfaction, and perceived productivity through work engagement. This hypothesis is in line with previous research reporting on benefits of work engagement for job satisfaction, and perceived productivity (Jurek & Besta, 2019; Kašpárková et al., 2018).

Our research is pre-registered and can be found on the Open Science Framework (OSF). To access the pre-registrations, please follow these links (Study 1: https://osf.io/3a25w/ and Study 2: https://osf.io/q4732/).

## Study 1

### Methods

#### Participants

We relied on previous research when determining sample size (Bouckenooghe and Raja, 2019) and collected data from 245 participants. Among the participants, 15 did not have a full-time job and 24 filled out only demographic variables. Therefore, they were excluded from further analyses. Furthermore, we excluded 7 outliers, who reported only extremely high or low on means of variables used to compute latent profiles. The final study was composed of 199 respondents (77 females). Age ranged from 18 to 65, with 60% of the participants being at the age ranges between 25 to 34. In addition, 51.26% of participants reported having a masters’ degree followed by 24.12% reporting having a university degree. Moreover, 61.81% of respondents were married and 34.67% single. Most of the participants reported having no children (41.71%), one child (29.65%), or two children (25.13%). Finally, respondents were living alone (30.65%), with parents/siblings (35.18%), or in a shared flat (31.66%).

#### Measures

##### Self-Regulation

Self-regulation was measured using the self-regulation subscale of the short version of the Volitional Components Inventory by Kuhl and Fuhrmann (2004). The subscale includes 12 items and participants are asked to rate the extent to which each item applies to them using a 4-point Likert-type scale ranging from 1 (*Not at all*) to 4 (*Completely*). A sample item is “I feel that most of the time I really want to do the things I do”. Cronbach’s alpha was .87.

##### Conscientiousness and Extraversion

Conscientiousness and extraversion were measured using 10-item version of the Big Five Inventory (Rammstedt & John, 2007), using 2 items for each. Participants were asked to indicate the extent to which each item describes their personality on a 5-point Likert-type scale ranging from 1 (*Strongly disagree*) to 5 (*Strongly agree*). Sample items for conscientiousness and extraversion are “… does a thorough job” and “… is outgoing, sociable” respectively. The scale showed good psychometric properties (Rammstedt & John, 2007).

##### Need for Autonomy

Need for autonomy was measured using seven items from Basic Psychological Need Satisfaction Short Scale (BNSG-S; Johnston & Finney, 2010). Participants were asked to indicate how true or untrue each item is for them using a 7-point Likert-type scale ranging from 1 (*Not at all true*) to 7 (*Very true*). A sample item is “I feel like I am free to decide for myself how to live my life”. Cronbach’s alpha was .67.

##### Job Satisfaction

The Job Satisfaction Survey (JSS; Spector, 1997) was used to measure job satisfaction. Participants were asked to indicate how satisfied they feel about different aspects of their job based on 13 items. These items target different aspects like salary, the chance of promotion, recognition, and working environment, which are likely to exert an influence on an individual’s levels of job satisfaction. A sample item is “I feel I am being paid a fair amount for the work I do”. Participants are asked to indicate to what extent they agree or disagree with each item on a scale from 1 (*Disagree very much*) to 6 (*Agree very much*). Cronbach’s alpha was .83.

##### Work Engagement

Work engagement was measured using the 18-item Job Engagement Scale (JES; Rich et al., 2010). Participants were asked to indicate the extent to which they agree or disagree with each item using a 5-point Likert-type scale ranging from 1 (*Strongly disagree*) to 5 (*Strongly agree*). A sample item includes “I work with intensity on my job”. Cronbach’s alpha was .92.

##### Perceived Productivity

Perceived productivity was measured using a question we generated. Participants were asked to indicate the extent to which they felt productive or unproductive when thinking about their work for the past week using a 7-point Likert-type scale ranging from 1 (*Completely unproductive*) to 7 (*Completely productive*).

##### Telecommuting Preference

Telecommuting preference was measured using a question we generated. Participants were asked for their preference of working from home (1) or on-site (0), everything else being equal (i.e., salary, benefits, position, etc.).

#### Statistical Analyses

##### Latent Profile Analyses

Using standardized mean values from variables and maximum likelihood estimator (MLR), a latent profile analysis with the tidyLPA R package (Rosenberg et al., 2018) was carried out to identify the best profile solution, freely estimating one to eight profiles in terms of means and variances. The optimal number of profiles was chosen based on the statistical adequacy of the solution, as well as fit indices (Nylund et al., 2007). Akaike Information Criterion (AIC), the Bayesian information criterion (BIC), the sample-adjusted BIC (ABIC), and the Bootstrap Likelihood Ratio Test (BLRT) were used to decide the final profile solutions. Lower values on the AIC, BIC, and ABIC suggest better-fitting solutions (Ram & Grimm, 2009), with significant BLRT values.

##### Logistic Regression

Logistic regression was run to predict the telecommuting preferences of individuals based on their profile membership. In this analysis, telecommuting preference (0 = work on-site; 1 = work from home) was used as an outcome variable, profiles as predictors and the telecommutability of job, marital status, number of children, gender, education completed, and living status as control variables.

##### Mediation Analyses

Two mediation analyses were run to test the hypotheses that work engagement mediates the relationship between profiles, job satisfaction, and perceived productivity. In these analyses, profiles were used as predictors, work engagement as a mediator, and job satisfaction and perceived productivity as outcome variables. To test the proposed mediation hypotheses, we used Preacher and Hayes' (2008) bootstrapping approach with a total of 5000 resamples and 95% confidence intervals. In these analyses, we set profile 1 as the reference category. The analyses were conducted using SPSS PROCESS tool (Hayes, 2020).

### Results

Descriptive statistics and correlations are presented in Table 1 in supplementary materials.

#### Number of Profiles (Pre-Registered, Confirmatory)

**Fig. 1:**
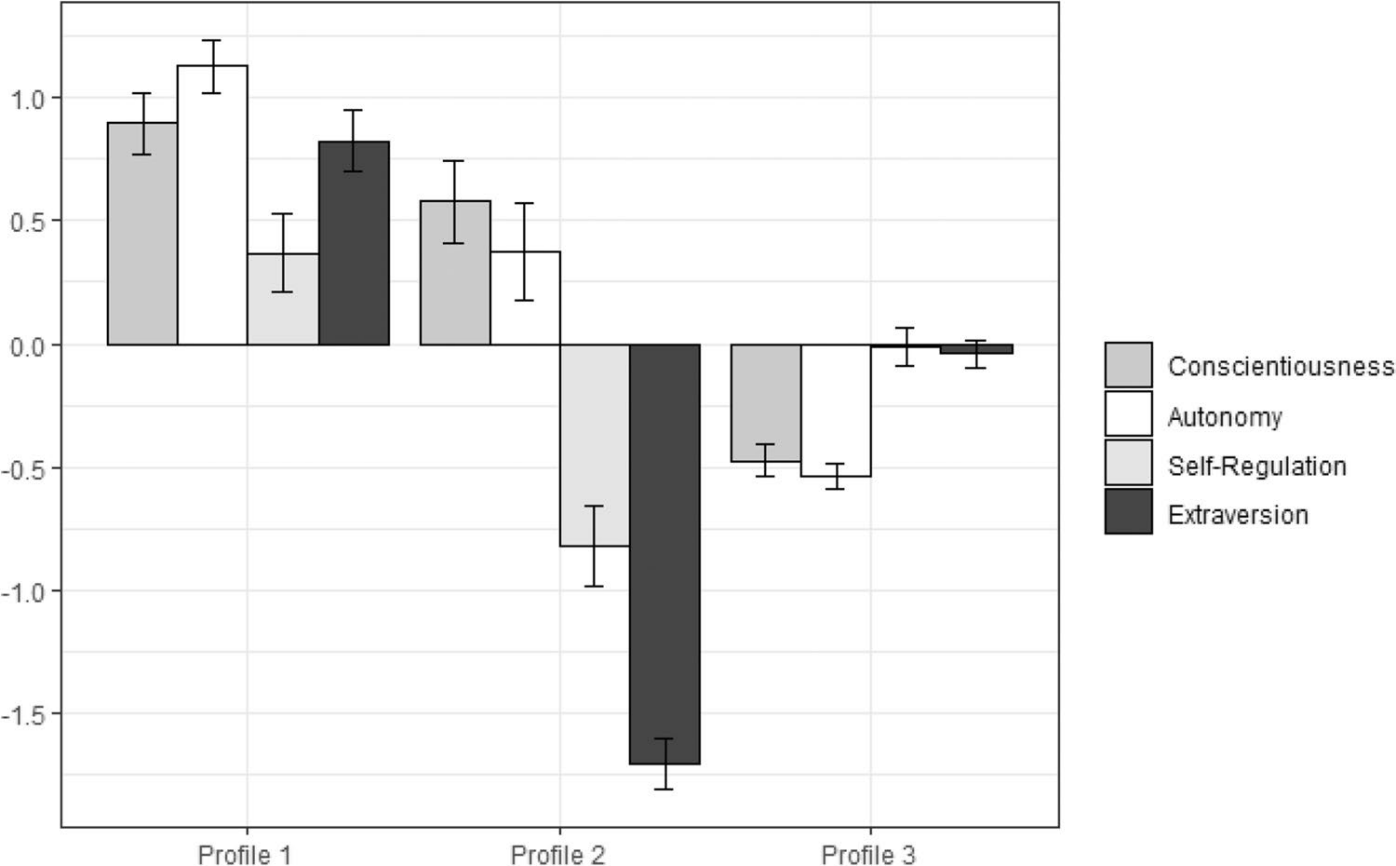
Characteristics of profiles (Study 1). Indicators are standardized with a mean of 0 and standard deviation of 1. Error bars represent standard errors. Profile 1: *high conscientious and autonomous, and medium-high extravert and self-regulated*; Profile 2: *medium-high conscientious and autonomous, and low extravert and medium-low self-regulated*; Profile 3: *medium-low self-regulated, conscientious, autonomous, extravert*

A three-profile solution was decided based on fit indices (see Table 1). As shown in Fig. 1, profile 1 (26.13%) displayed high scores on conscientiousness and autonomy, and medium-high on extraversion and self-regulation. Profile 2 (11.06%) showed medium-high on conscientiousness and autonomy, low on extraversion, and medium-low on self-regulation. Profile 3 (62.81%) demonstrated medium-low scores on self-regulation, conscientiousness, autonomy, and extraversion.

**Table 1:** Fit indices and number of profiles for Study 1 and Study 2

# of Profiles	LL	AIC	BIC	SABIC	BLRT(p)	Entropy
*Study 1*						
1	−839.86	1707.72	1753.82	1709.47	–	1.00
2	−817.01	1672.03	1734.60	1674.41	0.01	0.86
3	−790.12	**1628.23**	**1707.27**	**1631.24**	0.01	**0.90**
4	−788.69	1635.38	1730.88	1639.01	0.75	0.73
*Study 2*						
1	−2151.44	4330.88	4389.66	4345.22	–	1.00
2	−2109.71	4257.42	4337.19	4276.88	0.01	0.79
3	−2068.22	4184.44	4285.20	4209.03	0.01	0.87
4	−2034.91	4127.81	4249.57	4157.52	0.01	0.86
5	−2010.02	4088.04	4230.79	4122.87	0.01	0.69
6	−1967.40	**4012.80**	**4176.54**	**4052.76**	0.01	**0.88**
7	−1967.71	4023.43	4208.16	4068.50	0.99	0.80

*Notes*: Bold font indicates selected models. Study 1, *n* = 199; Study 2, *n* = 492. LL = log likelihood; AIC = Akaike information criteria; BIC = Bayesian information criteria; SABIC = sample-size adjusted BIC; BLRT = bootstrapped log-likelihood ratio test

Furthermore, we investigated the correlates of profiles and compared the profiles based on job telecommutability, perceived productivity, job satisfaction, and work engagement. Pairwise comparisons did not yield any significant difference between profiles in terms of job telecommutability. For more information, see Table 2 in the supplementary materials.

#### Logistic Regression (Pre-Registered, Confirmatory)

The results of logistic regression showed that compared to individuals in profile 1 (*high conscientious, autonomous, and medium-high extravert, self-regulated*), individuals in both profile 2 (*medium-high conscientious, autonomous, and low extravert and medium-low self-regulated*) and profile 3 (*medium-low self-regulated, conscientious, autonomous, extravert*) reported higher telecommuting preferences, *b* = 1.85, Wald χ^2^(1) = 7.03, *p* < .01, *OR* = 6.34 (95% CI [5.66, 7.11]) and *b* = 2.12, Wald χ^2^(1) = 18.80, *p* < .001, *OR* = 8.35 (95% CI [7.89, 8.83]). Complete results are provided in Table 3 in the supplementary materials.

#### Mediation Analyses (Pre-Registered, Confirmatory)

The results demonstrated a significant relative total and indirect effect (through work engagement) of profile 2 (*medium-high conscientious, autonomous, and low extravert, and medium-low self-regulated* vs. profile 1, *high conscientious, autonomous, and medium-high extravert, self-regulated*), profile 3 (*medium-low self-regulated, conscientious, autonomous, extravert* vs. profile 1, *high conscientious, autonomous, and medium-high extravert, self-regulated*) on job satisfaction. Moreover, a significant relative total effect of profile 2 (*medium-high conscientious, autonomous, and low extravert and medium-low self-regulated* vs. profile 1, *high conscientious, autonomous, and medium-high extravert, self-regulated*) but not of profile 3 (*medium-low self-regulated, conscientious, autonomous, extravert* vs. profile 1, *high conscientious, autonomous, and medium-high extravert, self-regulated*) and significant relative indirect effects (through work engagement) of profile 2 (*medium-high conscientious, autonomous, and low extravert and medium-low self-regulated* vs. profile 1, *high conscientious, autonomous, and medium-high extravert, self-regulated*) and profile 3 (*medium-low self-regulated, conscientious, autonomous, extravert* vs. profile 1, *high conscientious, autonomous, and medium-high extravert, self-regulated*) on perceived productivity were reported. For more details, see Table 2.

**Table 2:** Relative direct, indirect and total effects of profiles (and work engagement) on work engagement, job satisfaction and perceived productivity

Paths	Work Engagement	Job satisfaction	Perceived Productivity
*Individual paths*			
Profile 2 (vs. Profile 1)	a = −0.33*	*c’* = −0.46**	*c’* = −0.86*
Profile 3 (vs. Profile 1)	a = −0.37**	*c’* = −0.60**	*c’* = 0.05
Work Engagement		*b* = 0.34**	*b* = 0.92**
*Relative indirect effect*			
Profile 2 (vs. Profile 1)		*ab* = −0.11, *SE* = 0.06, *95% CI* [−0.25, −0.01]	*ab* = −0.31, *SE* = 0.15, *95% CI* [−0.62, −0.02]
Profile 3 (vs. Profile 1)		*ab* = −0.12, *SE* = 0.04, *95% CI* [−0.21, −0.05]	*ab* = −0.34, *SE* = 0.09, *95% CI* [−0.52, −0.16]
*Relative total effect*			
Profile 2 (vs. Profile 1)		*c* = −0.57**, *SE* = 0.16, *95% CI* [−0.89, −0.26]	*c* = −1.17, *SE* = 0.31, *95% CI* [−1.78, −0.56]
Profile 3 (vs. Profile 1)		*c* = −0.72, *SE* = 0.10, *95% CI* [−0.93, −0.52]	*c* = −0.29, *SE* = 0.20, *95% CI* [−0.69, 0.10]

*CI* = Confidence Interval. *a* indicates the path from predictors to mediator, *b* indicates the path from mediator to outcome variable, *c’* indicates the direct effect of predictor on outcome variable after controlling for the effect of mediator, *ab* indicates the indirect effect of predictor on outcome variable through mediator and *c* indicates the direct effect of predictor on outcome variable. Profile 1: *high conscientiousness and autonomy, and medium-high extraversion and self-regulation*; Profile 2: medium-high *conscientiousness and autonomy, and low extraversion and medium-low self-regulation*; Profile 3: medium-low *self-regulation, conscientiousness, autonomy, extraversion.* * indicates *p* < .05. ** indicates *p* < .001

## Study 2

To validate the findings of our Study 1, we conducted Study 2. Our rationale was based on recent calls reporting larger samples resulting in higher accuracy when identifying the appropriate number of latent profiles (Spurk et al., 2020). In their review research, Spurk et al. (2020) reported that “a minimum sample size of about 500 should lead to enough accuracy in identifying a correct number of latent profiles” (p. 6). Thus, to validate our Study 1, we collected data in a second term using Amazon Mechanical Turk (MTurk) and targeted a sample of over 500 participants. MTurk has been widely used in organizational research, reported to yield high-quality data, comparable to data collected in-person (Aguinis et al., 2021; Bennett et al., 2016; Buhrmester et al., 2011).

### Methods

#### Participants

In this study, we collected data from 539 participants. Among these participants, 23 did not have a full-time job, 6 filled out only demographic variables and thus were excluded from further analyses. Moreover, we excluded 19 outliers, who reported only high or low means of variables used to compute latent profiles. The final study consisted of 492 respondents (167 females). Age ranged from 18 to 65, with more than 60% of the participants being at the age ranges between 25 to 34. Additionally, 35.37% of participants had a masters’ degree, followed by 31.10% with a university degree. Furthermore, 62.60% of respondents were married and 31.50% single. In addition, most of the participants had no children (34.96%), one child (34.96%), or two children (22.97%). The majority lived alone (36.38%), with parents/siblings (40.85%), or in a shared flat (21.34%).

Measures and statistical analyses were the same as in the Study 1. Cronbach’s alpha for self-regulation (α = .87), autonomy (α = .64), work engagement (α = .93), and job satisfaction (α = .80) were relatively similar to Study 1.

### Results

Descriptive statistics and correlations are presented in Table 4 in supplementary materials.

#### Number of Profiles (Pre-Registered, Confirmatory)

**Fig. 2:**
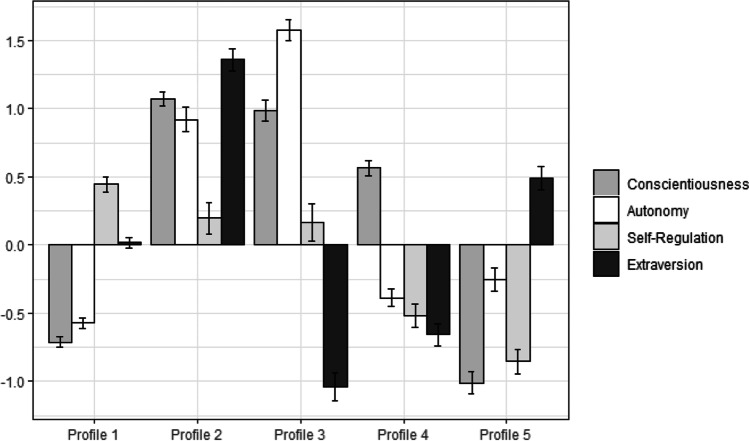
Characteristics of profiles (Study 2). Indicators are standardized with a mean of 0 and standard deviation of 1. Error bars represent standard errors. Profile 1: *medium-low conscientious, autonomous, and medium-high extravert, self-regulated;* Profile 2: *high extravert, conscientious, autonomous, and medium-high self-regulated*; Profile 3: *high conscientious, autonomous, medium-high self-regulated, and low extravert;* Profile 4: *medium-high conscientious, and medium-low self-regulated, autonomous, extravert*; Profile 5: *low conscientious, medium-low self-regulated, autonomous, and medium-high extravert*

Based on the fit indices presented in Table 1, a six-profile solution was optimal. However, as the lowest profile included less than 25 cases, we retained a solution with 5 profiles (Spurk et al., 2020), As shown in Fig. 2, profile 1 (38.42%) scored medium-low on conscientiousness and autonomy, and medium-low on extraversion and self-regulation. Profile 2 (14.84%) scored high on extraversion, conscientiousness, and autonomy and medium-high on self-regulation. Profile 3 (12.60%) scored high on conscientiousness and autonomy, medium-high on self-regulation, and low on extraversion. Profile 4 (21.34%) scored medium-high on conscientiousness, and medium-low on self-regulation, autonomy and extraversion. Finally, profile 5 (12.80%) scored low on conscientiousness, medium-low self-regulation and autonomy and medium-high extraversion.

Furthermore, we investigated the correlates of profiles and compared the profiles based on telecommutability of jobs, perceived productivity, job satisfaction and work engagement. Pairwise comparisons yielded a significant difference between profiles in terms of job telecommutability. Specifically, individuals in profile 1 (*medium-low conscientious, autonomous, and medium-high extravert, self-regulated*) rated their jobs to be significantly more telecommutable than those in profiles 3 (*high conscientious, autonomous, medium-high self-regulated, and low extravert*), 4 (*medium-high conscientious, and medium-low self-regulated, autonomous, extravert*) and 5 (*low conscientious, medium-low self-regulated, autonomous, and medium-high extravert*). For more details, see Table 5 in the supplementary materials.

#### Logistic Regression (Pre-Registered, Confirmatory)

To examine differences in profile membership, in terms of telecommuting preferences, we computed a logistic regression. Profile 2 (*high extravert, conscientious, autonomous, and medium-high self-regulated*) was chosen as the reference category. The results showed that compared to individuals in profile 2 (*high extravert, conscientious, autonomous, and medium-high self-regulated*), individuals in both profile 1 (*medium-low conscientious, autonomous, and medium-high extravert, self-regulated; b* = 1.18, Wald χ^2^(1) = 6.00, *p* < .05, *OR* = 3.26 (95% CI [3.00, 3.54]) and profile 5 (*low conscientious, medium-low self-regulated, autonomous, and medium-high extravert; b* = 1.44, Wald χ^2^(1) = 9.20, *p* < .01, *OR* = 4.21 (95% CI [3.42, 5.17]), reported higher telecommuting preferences. However, individuals in profile 3 (*high conscientious, autonomous, medium-high self-regulated, and low extravert*) and profile 4 (*medium-high conscientious, and medium-low self-regulated, autonomous, extravert*) did not report higher telecommuting preferences compared to profile 2 (*high extravert, conscientious, autonomous, and medium-high self-regulated*) *b* = 0.59, Wald χ^2^(1) = 1.80, *p* > .05, *OR* = 1.81 (95% CI [1.67, 1.96]), and *b* = 0.57, Wald χ^2^(1) = 2.20, *p* > .05, *OR* = 1.76 (95% CI [1.74, 1.79]) respectively. Complete results are provided in Table 6 in the supplementary materials.

#### Mediation Analyses (Pre-Registered, Confirmatory)

For the mediation analyses, we followed the same procedure as in Study 1. We set the profile 2 (*high extravert, conscientious, autonomous, and medium-high self-regulated*) as the reference category. The analyses demonstrated a significant relative total and indirect effect of profile 1 (*medium-low conscientious, autonomous, and medium-high extravert, self-regulated*; vs. profile 2, *high extravert, conscientious, autonomous, and medium-high self-regulated*), profile 4 (*medium-high conscientious, and medium-low self-regulated, autonomous, extravert*; vs. profile 2, *high extravert, conscientious, autonomous, and medium-high self-regulated*) and profile 5 (*low conscientious, medium-low self-regulated, autonomous, and medium-high extravert* vs. profile 2, *high extravert, conscientious, autonomous, and medium-high self-regulated*) on job satisfaction. Moreover, the analyses showed a significant relative total effect of profile 1 (*medium-low conscientious, autonomous, and medium-high extravert, self-regulated*; vs. profile 2, *high extravert, conscientious, autonomous, and medium-high self-regulated*), profile 4 (*medium-high conscientious, and medium-low self-regulated, autonomous, extravert*; vs. profile 2, *high extravert, conscientious, autonomous, and medium-high self-regulated*) and profile 5 (*low conscientious, medium-low self-regulated, autonomous, and medium-high extravert* vs. profile 2, *high extravert, conscientious, autonomous, and medium-high self-regulated*) on perceived productivity and a significant relative indirect effect of profile 1 (*medium-low conscientious, autonomous, and medium-high extravert, self-regulated* vs. profile 2, *high extravert, conscientious, autonomous, and medium-high self-regulated*), profile 3 (*high conscientious, autonomous, medium-high self-regulated, and low extravert*; vs. profile 2, *high extravert, conscientious, autonomous, and medium-high self-regulated*), profile 4 (*medium-high conscientious, and medium-low self-regulated, autonomous, extravert* vs. profile 2, *high extravert, conscientious, autonomous, and medium-high self-regulated*) and profile 5 (*low conscientious, medium-low self-regulated, autonomous, and medium-high extravert* vs. profile 2, *high extravert, conscientious, autonomous, and medium-high self-regulated*) on perceived productivity. Results are summarized in Table 3.

**Table 3:** Relative direct, indirect and total effects of profiles (and work engagement) on work engagement, job satisfaction and perceived productivity

Paths	Work Engagement	Job satisfaction	Perceived Productivity
*Individual paths*			
Profile 1 (vs. Profile 2)	*a* = −0.22*	*c’* = −0.73**	*c’* = 0.35*
Profile 3 (vs. Profile 2)	*a* = −0.10	*c’* = 0.14	*c’* = −0.01
Profile 4 (vs. Profile 2)	*a* = −0.42**	*c’* = −0.62**	*c’* = −0.05
Profile 5 (vs. Profile 2)	*a* = −0.73**	*c’* = −0.47**	*c’* = −0.23
Work Engagement		*b* = 0.32**	*b* = 0.78**
*Relative indirect effect*			
Profile 1 (vs. Profile 2)		*ab* = −0.07, *SE* = 0.03, *95% CI* [−0.13, −0.02]	*ab* = −0.17, *SE* = 0.06, *95% CI* [−0.30, −0.06]
Profile 3 (vs. Profile 2)		*ab* = −0.03, *SE* = 0.03, *95% CI* [−0.10, 0.03]	*ab* = −0.08, *SE* = 0.08, *95% CI* [−0.24, −0.07]
Profile 4 (vs. Profile 2)		*ab* = −0.14, *SE* = 0.04, *95% CI* [−0.22, −0.07]	*ab* = −0.33, *SE* = 0.08, *95% CI* [−0.49, −0.19]
Profile 5 (vs. Profile 2)		*ab* = −0.23, *SE* = 0.06, *95% CI* [−0.35, −0.14]	*ab* = −0.57, *SE* = 0.11, *95% CI* [−0.80, −0.37]
*Relative total effect*			
Profile 1 (vs. Profile 2)		c = −0.80**, *SE* = 0.09, *95% CI* [−0.97, −0.62]	c = 0.18, *SE* = 0.18, *95% CI* [−0.08, 0.45]
Profile 3 (vs. Profile 2)		c = 0.11, *SE* = 0.11, *95% CI* [−0.10, 0.33]	c = −0.08, *SE* = 0.17, *95% CI* [−0.41, 0.25]
Profile 4 (vs. Profile 2)		c = −0.76**, *SE* = 0.10, *95% CI* [−0.95, −0.57]	c = −0.38*, *SE* = 0.15, *95% CI* [−0.67, −0.09]
Profile 5 (vs. Profile 2)		c = −0.71**, *SE* = 0.11, *95% CI* [−0.92, −0.49]	c = −0.80**, *SE* = 0.17, *95% CI* [−1.13, −0.47]

*CI* = Confidence Interval. *a* indicates the path from predictors to mediator, *b* indicates the path from mediator to outcome variable, *c’* indicates the direct effect of predictor on outcome variable after controlling for the effect of mediator, *ab* indicates the indirect effect of predictor on outcome variable through mediator and *c* indicates the direct effect of predictor on outcome variable. Profile 1: *medium-low conscientious, autonomous, and medium-high extravert, self-regulated;* Profile 2: *high extravert, conscientious, autonomous, and medium-high self-regulated*; Profile 3: *high conscientious, autonomous, medium-high self-regulated and low extravert;* Profile 4: *medium-high conscientious and medium-low self-regulated, autonomous, extravert*; Profile 5: *low conscientious, medium-low self-regulated, autonomous, and medium-high extravert.* * indicates *p* < .05. ** indicates *p* < .001

## General Discussion

With this two-study research, we aimed at investigating the preferences of employees for telecommuting. By collecting data during the first wave of COVID-19, we add to the wealth of research during the crisis. To investigate telecommuting preferences, we used various motivational (i.e., autonomy and self-regulation) and personality (i.e., conscientiousness and extraversion) constructs to create employee profiles. Furthermore, we examined work-related correlates of profiles, namely work engagement, job satisfaction, and perceived productivity.

In Study 1, our findings revealed three profiles. One profile scored high on all four CASE variables. Contrary to our expectations, profile 1 (*high conscientious, autonomous, and medium-high extravert, self-regulated*) showed lower telecommuting preferences, compared to profile 2 (*medium-high conscientious, autonomous, and low extravert and medium-low self-regulated*) and 3 (medium-low *self-regulated, conscientious, autonomous, extravert*). However, in line with our expectations, profile 1 (*high conscientious, autonomous, and medium-high extravert, self-regulated*) reported high work engagement, job satisfaction, and perceived productivity, compared to other profiles. This finding is in line with previous research demonstrating the positive relationships between CASE variables and work engagement, job satisfaction, and perceived productivity (e.g., Barrick & Mount, 1991; Morgeson & Humphrey, 2008). Furthermore, mediation analyses revealed a significant relative indirect effect of profile 2 (*medium-high conscientious, autonomous, and low extravert and medium-low self-regulated* vs. profile 1, *high conscientious, autonomous, and medium-high extravert, self-regulated*) and profile 3 (*medium-low self-regulated, conscientious, autonomous, extravert* vs. profile 1, *high conscientious, autonomous, and medium-high extravert, self-regulated*) on both job satisfaction and perceived productivity through work engagement (see Table 2). Accounting for previous research and given the scores of profile 2 (*medium-high conscientious, autonomous, and low extravert and medium-low self-regulated)* and profile 3 (*medium-low self-regulated, conscientious, autonomous, extravert*) on all four CASE constructs, these results were expected (Barsness et al., 2005; Blickle et al., 2015).

In Study 2, which we used as a validation of the Study 1 findings, results revealed two more profiles. Three profiles were relatively similar to Study 1 in terms of their values in the CASE variables. Similar to Study 1, profile 2 (*high extravert, conscientious, autonomous, and medium-high self-regulated*) demonstrated lower telecommuting preferences than profile 1 (*medium-low conscientious, autonomous, and medium-high extravert, self-regulated*) and profile 5 (*low conscientious, medium-low self-regulated, autonomous, and medium-high extravert*), but not different from profile 3 (*high conscientious, autonomous, medium-high self-regulated, and low extravert*) and profile 4 (*medium-high conscientious, and medium-low self-regulated, autonomous, extravert*). Furthermore, similar to the findings in Study 1, profile 2 (*high extravert, conscientious, autonomous, and medium-high self-regulated*) reported higher work engagement, job satisfaction, and perceived productivity, compared to profile 1 (*medium-low conscientious, autonomous, and medium-high extravert, self-regulated*), profile 4 (*medium-high conscientious, and medium-low self-regulated, autonomous, extravert*), and profile 5 (*low conscientious, medium-low self-regulated, autonomous, and medium-high extravert*). Analogous to Study 1, mediation analyses revealed a significant relative indirect effect of profile 1 (*medium-low conscientious, autonomous, and medium-high extravert, self-regulated* vs. profile 2), profile 4 (*medium-high conscientious, and medium-low self-regulated, autonomous, extravert* vs. profile 2, *high extravert, conscientious, autonomous, and medium-high self-regulated*) and profile 5 (*low conscientious, medium-low self-regulated, autonomous, and medium-high extravert* vs. profile 2, *high extravert, conscientious, autonomous, and medium-high self-regulated*) on both job satisfaction and perceived productivity through work engagement. However, profile 3 (*high conscientious, autonomous, medium-high self-regulated, and low extravert*) and profile 2 (*high extravert, conscientious, autonomous, and medium-high self-regulated*) did not differ on job satisfaction but was significantly related to perceived productivity through work engagement. See Table 3 for more details. Similar to the findings in Study 1, the results of Study 2 are also in line with previous research showing the benefits of individuals who score high on CASE variables in relation to work engagement, job satisfaction, and perceived productivity (e.g., Barrick & Mount, 1991; Morgeson & Humphrey, 2008).

Overall, our findings might imply that individuals who *vary* among CASE variables (*high and low*) might indeed benefit from a telecommuting context. In line with previous research (Chang et al., 2020; Leslie et al., 2012), this might also reveal implications with employees’ work-related outcomes, such as work engagement or job satisfaction. Because telecommuting is reported to challenge employees’ regular work schedules, providing employees (i.e., those scoring high and low in CASE variables) with ways of enhancing their personality and motivational traits, might indeed be beneficial in improving their work engagement and productivity, as well as job satisfaction. According to the JD-R-model (Demerouti et al., 2001), a balance between job demands and resources, is an effective way to mitigate the negative influences of various work-related strains. Thus, it might be important for organizations to reduce demands yielded by telecommuting through the provision of a supportive environment, such as through continuous feedback (Timms et al., 2015) or facilitating the transition between an office and home environment (Galanti et al., 2021). For example, because extraverts are more sociable and talkative (Costa & McCrae, 1992), it might be important that organizations provide more feedback to this type of employee, which would lessen the negative effects of isolation brought by telecommuting. However, this might not be the case for autonomous employees, due to their preferences for more independent and less supervised work conditions (Konradt et al., 2003). However, for individuals who scored *high* on all CASE variables, telecommuting might not be a “first-choice” preference. Nevertheless, the fact that these profiles (both for Study 1 and 2) reported higher work engagement, job satisfaction, and perceived productivity, might signify that, when they are faced with difficult choices at work (such as telecommuting), they might produce better work results, beneficial for both, individuals and organizations, at large. Our findings are in line with previous research reporting that CASE variables, such as job autonomy (e.g., Konradt et al., 2003) and conscientiousness (e.g., Haines et al., 2002) are associated with positive telecommuting attitudes. Thus, in accordance with the organizational fit theory (Judge & Cable, 1997; Ryan & Kristof-Brown, 2003), our findings imply that organizations might support employees’ adaptation to new environments (i.e., working from home) by providing support based on the “type” of the motivational and personality traits. For instance, accounting that conscientious employees are more organized and self-disciplined (McCrae & Costa, 2003), our findings signify that this type (scoring high on both studies) might indeed prefer fewer interactions and more autonomy for better work-related outcomes, such as work engagement and job productivity, as well as job satisfaction. Hence, for such personality types, this form of communication and interaction might yield better outcomes in a telecommuting environment. Similar implications can be drawn with individuals with a high level of self-regulation (Beal et al., 2005) and job autonomy (Ryan & Deci, 2000). According to the Beal et al.’ (2005) model of self-regulation, balancing between task and off-task demands, might yield benefits for employees with high self-regulatory mechanisms. Therefore, since individuals who scored high on this trait, also reported higher work engagement, job productivity, and job satisfaction in our research, might be of crucial importance that organizations pay attention to the demands placed on these types of employees by reducing the load of “unnecessary” tasks and focus on the provision of tasks and support (i.e., feedback) that would facilitate their growth in a telecommuting environment.

To our knowledge, our research is the first of its kind to study telecommuting preferences by using CASE variables, through latent profile analysis, especially during a pandemic context. Previous studies have mainly used variable-centered approaches. We, however, used a person-centered approach, which is reported to provide a stronger and more in-depth approach for investigating the interaction of the different motivational and personality factors (Morin & Wang, 2016), “allowing researchers to understand how variables operate conjointly and within people to shape outcomes” (Gabriel et al., 2015, p. 865). Furthermore, similar to our study, there is a large body of research currently reporting on the implications that various motivational and psychological constructs have on telecommuting preferences and work-related outcomes. For example, current studies reported that factors such as work motivation (e.g., Mahmoud et al., 2020) or perceived organizational support and psychological safety (e.g., Lee, 2021) and similar variables are also important to consider for employees’ telecommuting preferences and work outcomes. Nevertheless, these studies have mostly employed a variable-centered approach. Our research adds to that by investigating work-related outcomes by person-centered approaches using other motivational and personality factors.

### Theoretical and Practical Implications, Limitations, and Future Research

Implications of our findings are manifold. Theoretically, our findings reveal that accounting for the importance of the CASE variables for telecommuting preferences in this research, theories such as Big Five (Allport & Odbert, 1936) or Self-Determination (SDT; Deci & Ryan, 1985), might consider the importance of variations in scores among individuals. For example, SDT has long reported on the importance of autonomy as a crucial factor for one’s need to feel competent and related to others. Thus, the relatedness facet might interfere with employees’ performance in a telecommuting context, which in turn will influence their preferences and attitudes towards this form of work (Deci & Vansteenkiste, 2004). Nevertheless, this was not fully the case with our findings. Although telecommuting preferences did vary among profiles, there was a different result with work-related outcomes. For instance, in Study 2, the logistic regression analysis revealed that profile 1 (*medium-low conscientious, autonomous, and medium-high extravert, self-regulated)* and profile 5 (*low conscientious, medium-low self-regulated, autonomous, and medium-high extravert*) demonstrated the highest preferences towards telecommuting preferences. As shown, both of these profiles scored medium-low in the key facet of the SDT, namely autonomy. Alluding to SDT claims (e.g., Deci & Ryan, 1985; Ryan & Deci, 2000), one would expect a different result, because findings from the SDT have asserted that high autonomous individuals might benefit from this feature in challenging environments. Thus, we expected that individuals reporting low autonomy would show a lower tendency on their telecommuting preferences. However, one crucial claim of the SDT is that individuals might benefit from challenging environments only when they offer room for individuals to feel “competent, related, and autonomous” (Ryan & Deci, 2000, p. 74). Therefore, it might be that individuals in these profiles reported high telecommuting preferences because of their supportive environment. This claim is in line with current research findings. For example, Lee (2021) reported that, among others, one’s perceived organizational support (i.e., organizations care for employees’ well-being and contributions) plays an important role for employees’ psychological safety and well-being during a health crisis. These findings indicate that, similar to SDT claims, a supportive climate in organizations, can be beneficial for employees’ telecommuting preferences. In these similar assertions might the interpretation of our further findings, where, contrary to profiles above, profile 2 (*high extravert, conscientious, autonomous, and medium-high self-regulated*) and profile 3 (*high conscientious, autonomous, medium-high self-regulated, and low extravert*), in Study 2, showed low preferences towards telecommuting. In other words, employees’ level of autonomy might be insignificant in relations to employees’ telecommuting preferences (as well as benefits), if organizations provide no support. Thus, our findings add to the important claims that theories such as the SDT assert and the importance that a supportive environment might play for employees’ functioning in a health crisis and challenging environments such as COVID-19. Nevertheless, as our results revealed, these telecommuting preferences do not necessarily translate into better work-related outcomes, where profile 1 (*medium-low conscientious, autonomous, and medium-high extravert, self-regulated*) and 5 (*low conscientious, medium-low self-regulated, autonomous, and medium-high extravert*), despite their high preferences for working from home, reported lower work-engagement, job satisfaction, and perceived productivity when doing so.

Practically, our findings offer many implications. On the one hand, our findings imply that individuals do not need to score high on all four CASE variables for a “best” type of employee profile, which would predict their preferences towards telecommuting. For instance, in our Study 2, profile 2 (*high extravert, conscientious, autonomous, and medium-high self-regulated*), showed lower telecommuting preferences, compared to profile 1 (*medium-low conscientious, autonomous, and medium-high extravert, self-regulated)* and profile 5 (*low conscientious, medium-low self-regulated, autonomous, and medium-high extravert*). Because in profile 2 (*high extravert, conscientious, autonomous, and medium-high self-regulated*) individuals reported to be high on extraversion, this might be one factor interfering with their telecommuting preferences. These results are also in line with previous research, theories, and findings. For example, extraverts are sociable and talkative (Barrick & Mount, 1991), and prefer an environment with social interactions, and therefore might suffer from crisis and environment with reduced social interactions. Previous research has shown that, although these individuals can benefit from telecommuting, here also, the environment plays a significant role. (e.g., Clark et al., 2012). According to Clark and colleagues, for extraverts to benefit from telecommuting, the environment should be enough stimulating and offer the possibility for interaction with their relatives. Therefore, our results might imply that individuals in this profile might have “suffered” from a poor environment, which in turn could have influenced their attitudes towards telecommuting. Thus, individuals should also consider the importance of these variations in the light of positive factors facilitating their work (i.e., telecommuting preferences) during challenging times such as COVID-19. On the other hand, our findings also draw implications for companies at large. Given the challenges that companies faced with their workforces during COVID-19, our findings might suggest that for companies to adapt to such situations (i.e., through telecommuting), they do not necessarily need to have the “ideal” employee (i.e., low in some features and high on others) or train them to be one. In contrast, our findings revealed that telecommuting can be both beneficial and a possible hindrance. For employees scoring high on all four CASE variables, telecommuting might be a hindrance. However, for the same individuals, it improves work-related outcomes. Furthermore, for employees scoring low on CASE variables, telecommuting might be beneficial. However, this does not guarantee the quality of their work performance, as revealed by our findings. Thus, organizations must act as facilitators during challenging times, such as health crises, and provide flexibility to their employees during their adjustments. While organizations might assume that employees high on autonomy (i.e., profile 2 in our Study 2, *high extravert, conscientious, autonomous, and medium-high self-regulated*) can adapt to changes and fit to new environments (such as moving from an on-site environment to an off-site setting), this might not always be the case. As our study revealed, this profile (which scored high on autonomy) showed low preferences towards telecommuting. However, although this kind of transition might be difficult for these kinds of profiles, they might benefit in relation to work-outcomes (where this profile expressed high work engagement, job satisfaction, and perceived productivity). Therefore, organizations should be supportive and provide room for flexibility, based on employee needs and other relevant motivational- and personality-characteristic.

## Limitations and Future Research

Our study has some limitations. First and foremost, our findings are purely correlational. Therefore, results cannot be interpreted as causal claims. Future longitudinal studies might add to the validation of this research. Secondly, data collection occurred during COVID-19,therefore this setting of our research needs to be accounted for whenever our findings are to be generalized. Future research (i.e., longitudinal research) might look at the implications that various contexts (i.e., during- and post-pandemic crisis) might yield for both LPA, as well as the currently used correlates (i.e., telecommuting preferences and work-related outcomes). Finally, we adhered to the recommendations of previous research when deciding on the sample of our Study 2 (e.g., Spurk et al., 2020). Thus, to further confirm and strengthen our findings, future research should employ a similarly large sample size. Similarly, future research should consider employing further variables in testing out conjectures. For example, it would be important to consider whether a supportive environment (as reported by SDT and other research) would have an impact on the telecommuting preferences of profile 2 (*high extravert, conscientious, autonomous, and medium-high self-regulated*) and profile 3 (*high conscientious, autonomous, medium-high self-regulated, and low extravert*) in our Study 2.

## Conclusion

In this two-study research, we adopted an intra-individual, person-centered approach, and investigated the telecommuting preferences of employees during COVID-19. In both of our studies, findings revealed a different number of profiles (3 profiles in Study 1; 5 profiles in Study 2). Furthermore, telecommuting preferences of employees varied across profiles. Specifically, profiles that scored high on all CASE variables, reported low telecommuting preferences, compared to profiles that scored low. However, these profiles reported higher work-related outcomes, compared to profiles that varied in their scores (i.e., high and low). Our research provides insights into the ramifications that various personality and motivational traits of employees play for their telecommuting preferences and work outcomes.

## Supplementary Information

ESM 1 (DOCX 28 kb)

## Data Availability

All materials and data are publicly accessible at https://osf.io/3a25w/ (for Study 1) and https://osf.io/q4732/ (for Study 2).

## Code Availability

Codes for data analyses are publicly accessible at https://osf.io/3a25w/ (for Study 1) and https://osf.io/q4732/ (for Study 2).
