# Supplementary material for: Predicting employee telecommuting preferences and job outcomes amid COVID-19 pandemic: a latent profile analysis
Source: Curr Psychol. 2021 Nov 19;42(10):8680–95. doi: 10.1007/s12144-021-02496-8 (PMC8602983; doi:10.1007/s12144-021-02496-8)
Supplement: Supplementary file 1 — (DOCX 28 kb) [file 12144_2021_2496_MOESM1_ESM.docx]

**Online Supplementary Materials for:**

**Predicting Telecommuting Preferences amid COVID-19 Pandemic: A Latent Profile Analysis**

**To be appeared in *Current Psychology***

Cafer Bakaç*, Jetmir Zyberaj, James C. Barela

^*^Corresponding author at: TUM School of Management, Chair of Psychology, Technical University of Munich, Arcisstraße 21 D-80333 München.

*E-mail address*: cafer.bakac@tum.de (Cafer Bakaç)

**Authors’ Note:**

| These supplementary materials are to provide readers with more information about results shortly mentioned in the main manuscript. They are to be posted on the journal’s website online and link to the manuscript. If the journal does not offer this possibility, the materials could be posted on open science framework website, where the studies had been pre-registered! |
| --- |

**Sections**

**Study 1**

1. Table 1. Means, standard deviations, and correlations

2. Table 2. Telecommuting preferences based on profiles and demographic variables

3. Table 3. Means of correlates and pairwise comparisons between the three profiles

**Study 2**

4. Table 4. Means, standard deviations, and correlations

5. Table 5. Telecommuting preferences based on profiles and demographic variables

6. Table 6. Means of correlates and pairwise comparisons between the five profiles

**Table 1**

*Means, standard deviations, and correlations (Study 1)*

| Variable | *M* | *SD* | 1 | 2 | 3 | 4 | 5 | 6 | 7 | 8 | 9 |
| --- | --- | --- | --- | --- | --- | --- | --- | --- | --- | --- | --- |
|  |  |  |  |  |  |  |  |  |  |  |  |
| 1. Gender (1 = Male) | 0.61 | 0.49 |  |  |  |  |  |  |  |  |  |
|  |  |  |  |  |  |  |  |  |  |  |  |
| 2. Job telecommutability | 7.84 | 2.48 | -.06 |  |  |  |  |  |  |  |  |
|  |  |  |  |  |  |  |  |  |  |  |  |
| 3. Perceived productivity | 5.63 | 1.25 | .16* | .19** |  |  |  |  |  |  |  |
|  |  |  |  |  |  |  |  |  |  |  |  |
| 4. Home office preference (1 = Home) | 0.72 | 0.45 | -.01 | .21** | .02 |  |  |  |  |  |  |
|  |  |  |  |  |  |  |  |  |  |  |  |
| 5. Self-regulation | 2.86 | 0.47 | .12 | .28** | .47** | -.04 |  |  |  |  |  |
|  |  |  |  |  |  |  |  |  |  |  |  |
| 6. Conscientiousness | 3.51 | 0.85 | .03 | -.06 | .12 | -.29** | .12 |  |  |  |  |
|  |  |  |  |  |  |  |  |  |  |  |  |
| 7. Extraversion | 3.04 | 0.83 | -.05 | .06 | .16* | -.22** | .21** | .08 |  |  |  |
|  |  |  |  |  |  |  |  |  |  |  |  |
| 8. Autonomy | 4.60 | 0.81 | -.16* | .10 | .14 | -.30** | .30** | .26** | .23** |  |  |
|  |  |  |  |  |  |  |  |  |  |  |  |
| 9. Job satisfaction | 3.84 | 0.70 | -.02 | .22** | .20** | -.24** | .35** | .27** | .18* | .55** |  |
|  |  |  |  |  |  |  |  |  |  |  |  |
| 10. Work engagement | 3.93 | 0.58 | .12 | .24** | .44** | -.11 | .61** | .37** | .13 | .32** | .38** |

*Note.* *M* and *SD* are used to represent mean and standard deviation, respectively. * indicates *p* < .05. ** indicates *p* < .01.

**Table 2**

*Telecommuting preference based on profiles and demographic variables (Study 1)*

| *Term* | *B(SE)* | *OR* |
| --- | --- | --- |
| Intercept | -2.82(1.62) | 0.06 |
| Profile 2 (vs. Profile 1) | 1.72(0.67)* | 5.58 |
| Profile 3 (vs. Profile 1) | 2.09(0.47)** | 8.11 |
| Marital status (Married vs. Divorced) | 2.15(1.37) | 8.60 |
| Marital status (Separated vs. Divorced) | 15.76(2399.55) | 7009880.71 |
| Marital status (Single vs. Divorced) | 0.42(1.42) | 1.53 |
| Marital status (Widowed vs. Divorced) | 17.73(2399.55) | 49892946.02 |
| Children (1 vs 0) | -0.16(0.65) | 0.85 |
| Children (2 vs 0) | -0.69(0.73) | 0.50 |
| Children (3 vs 0) | -0.60(1.27) | 0.55 |
| Children (4+ vs 0) | -18.20(2399.54) | 0.00 |
| Gender | -1.05(0.49)* | 0.35 |
| Job telecommutability | 0.19(0.08)* | 1.21 |
| Education completed (Higher education entrance vs. High school diploma) | 0.38(0.96) | 1.46 |
| Education completed (Still in school vs. High school diploma) | 0.93(3393.47) | 2.53 |
| Education completed (Vocational diploma vs. High school diploma) | 1.60(1.24) | 4.97 |
| Education completed (University vs. High school diploma) | 1.59(0.87) | 4.89 |
| Education completed (Master’s degree vs. High school diploma) | -0.03(0.78) | 0.97 |
| Education completed (Ph.D. vs. High school diploma) | 16.56(1619.17) | 15541887.99 |
| Education completed (Other vs. High school diploma) | -16.55(2399.54) | 0.00 |

*Note.* *R*^2^ = .28 (Cox & Snell), .41 (Nagelkerke), *N* = 199. OR = Odds Ratio. * *p* < .05. ** *p* < .01.

**Table 3**

*Means of correlates and pairwise comparisons between the three profiles (Study 1).*

| Correlates | Profile 1 | Profile 2 | Profile 3 | Differences between profiles |
| --- | --- | --- | --- | --- |
|  | Mean(SE) | Mean(SE) | Mean(SE) |  |
| Job telecommutability | 7.87 (1.30) | 7.50 (1.21) | 7.89 (2.81) | 1 = 2 = 3 |
| Perceived productivity | 5.94 (0.52) | 4.77 (0.60) | 5.65 (0.51) | 2 < 1 = 3 |
| Job satisfaction | 4.36 (0.44) | 3.79 (0.42) | 3.64 (0.22) | 2 = 3 < 1 |
| Work engagement | 4.20 (0.29) | 3.86 (0.33) | 3.83 (0.28) | 3 < 1; 1 = 2; 2 = 3 |

*Note.* *SE* is used to represent standard error.

**Table 4**

*Means, standard deviations, and correlations with confidence intervals (Study 2)*

| Variable | *M* | *SD* | 1 | 2 | 3 | 4 | 5 | 6 | 7 | 8 | 9 |
| --- | --- | --- | --- | --- | --- | --- | --- | --- | --- | --- | --- |
|  |  |  |  |  |  |  |  |  |  |  |  |
| 1. Gender (1 = Male) | 0.66 | 0.48 |  |  |  |  |  |  |  |  |  |
|  |  |  |  |  |  |  |  |  |  |  |  |
| 2. Job telecommutability | 8.02 | 2.36 | -.01 |  |  |  |  |  |  |  |  |
|  |  |  |  |  |  |  |  |  |  |  |  |
| 3. Perceived productivity | 5.96 | 1.02 | -.03 | .27** |  |  |  |  |  |  |  |
|  |  |  |  |  |  |  |  |  |  |  |  |
| 4. Home office preference (1 = Home) | 0.78 | 0.41 | -.08 | .36** | .08 |  |  |  |  |  |  |
|  |  |  |  |  |  |  |  |  |  |  |  |
| 5. Self-regulation | 2.95 | 0.51 | .06 | .20** | .39** | .03 |  |  |  |  |  |
|  |  |  |  |  |  |  |  |  |  |  |  |
| 6. Conscientiousness | 3.69 | 0.95 | -.15** | -.12* | .07 | -.20** | .10* |  |  |  |  |
|  |  |  |  |  |  |  |  |  |  |  |  |
| 7. Extraversion | 3.01 | 0.89 | .08 | -.09 | .14** | -.10* | .17** | .03 |  |  |  |
|  |  |  |  |  |  |  |  |  |  |  |  |
| 8. Autonomy | 4.62 | 0.77 | .00 | -.10* | .10* | -.10* | .31** | .49** | .07 |  |  |
|  |  |  |  |  |  |  |  |  |  |  |  |
| 9. Job satisfaction | 3.92 | 0.73 | .06 | -.05 | .20** | -.17** | .26** | .31** | .16** | .58** |  |
|  |  |  |  |  |  |  |  |  |  |  |  |
| 10. Work engagement | 4.08 | 0.59 | -.05 | .16** | .49** | -.06 | .57** | .27** | .17** | .30** | .33** |
|  |  |  |  |  |  |  |  |  |  |  |  |

*Note.* *M* and *SD* are used to represent mean and standard deviation, respectively. * indicates *p* < .05. ** indicates *p* < .01.

**Table 5**

*Telecommuting preference based on profiles and demographic variables (Study 2)*

| *Term* | *B(SE)* | *OR* |
| --- | --- | --- |
| Intercept | -4.09(1.69) | 0.02 |
| Profile 1 (vs. Profile 2) | 1.13(0.98)* | 3.09 |
| Profile 3 (vs. Profile 2) | 0.56(0.43) | 1.75 |
| Profile 4 (vs. Profile 2) | 0.54(0.38) | 1.71 |
| Profile 5 (vs. Profile 2) | 1.36(0.46)* | 3.91 |
| Marital status (Married vs. Divorced) | 0.63(0.63) | 1.88 |
| Marital status (Separated vs. Divorced) | 0.88(1.91) | 2.40 |
| Marital status (Single vs. Divorced) | 1.26(0.71) | 3.54 |
| Marital status (Widowed vs. Divorced) | -1.05(1.08) | 0.35 |
| Children (1 vs 0) | 0.87(0.43)* | 2.39 |
| Children (2 vs 0) | 0.17(0.44) | 1.19 |
| Children (3 vs 0) | 1.10(0.69) | 3.01 |
| Children (4+ vs 0) | -0.14(0.99) | 0.87 |
| Gender | -0.14(0.99)* | 0.49 |
| Job telecommutability | 0.30(0.05)* | 1.36 |
| Education completed (High school diploma vs. No qualifications) | 2.01(1.56) | 7.49 |
| Education completed (Higher education entrance vs. No qualifications) | 2.62(1.56) | 13.73 |
| Education completed (Junior high diploma vs. No qualifications) | 16.12(814.83) | 10042582.21 |
| Education completed (Still in school vs. No qualifications) | 14.81(1455.40) | 2694286.87 |
| Education completed (Vocational diploma vs. No qualifications) | 1.40(1.56) | 4.06 |
| Education completed (University vs. No qualifications) | 1.58(1.51) | 4.86 |
| Education completed (Master’s degree vs. No qualifications) | 1.42(1.52) | 4.15 |
| Education completed (Ph.D. vs. No qualifications) | 2.23(1.79) | 9.28 |
| Education completed (Other vs. No qualifications) | 0.90 (1.77) | 2.47 |

*Note.* *R*^2^ = .18 (Cox & Snell), .28 (Nagelkerke), *N* = 492. OR = Odds Ratio. * *p* < .05. ** *p* < .01.

**Table 6**

*Means of correlates and pairwise comparisons between the five profiles (Study 2)*

| Correlates | Profile 1 | Profile 2 | Profile 3 | Profile 4 | Profile 5 | Differences between profiles |
| --- | --- | --- | --- | --- | --- | --- |
|  | Mean(SE) | Mean(SE) | Mean(SE) | Mean(SE) | Mean(SE) |  |
| Job telecommutability | 8.93 (0.52) | 6.81 (1.21) | 7.79 (1.13) | 7.85 (0.92) | 7.25 (0.98) | 5 = 4 = 2 < 1; 1 = 3;  3 = 2 = 4 = 5 |
| Perceived productivity | 6.27 (0.37) | 6.08 (0.37) | 6.00 (0.40) | 5.70 (0.46) | 5.29 (0.46) | 5 < 1 = 2 = 3; 4 < 1;  2 = 3 = 4; 4 =5 |
| Job satisfaction | 3.67 (0.22) | 4.47 (0.38) | 4.58 (0.40) | 3.71 (0.37) | 3.76 (0.34) | 1 = 4 = 5 < 2 = 3 |
| Work engagement | 4.14 (0.27) | 4.36 (0.24) | 4.26 (0.29) | 3.94 (0.27) | 3.63 (0.33) | 5 < 4 < 1 = 2 = 3 |

*Note.* *SE* is used to represent standard error.
